# Effective Actions of Ion Release from Mesoporous Bioactive Glass and Macrophage Mediators on the Differentiation of Osteoprogenitor and Endothelial Progenitor Cells

**DOI:** 10.3390/pharmaceutics13081152

**Published:** 2021-07-27

**Authors:** Alberto Polo-Montalvo, Laura Casarrubios, María Concepción Serrano, Adrián Sanvicente, María José Feito, Daniel Arcos, María Teresa Portolés

**Affiliations:** 1 Departamento de Bioquímica y Biología Molecular, Facultad de Ciencias Químicas, Universidad Complutense de Madrid, Instituto de Investigación Sanitaria del Hospital Clínico San Carlos (IdISSC), 28040 Madrid, Spain; albpolo@ucm.es (A.P.-M.); laura.casarrubios.molina@gmail.com (L.C.); adriansanvicenteg@gmail.com (A.S.); mjfeito@ucm.es (M.J.F.); 2 Instituto de Ciencia de Materiales de Madrid (ICMM), Consejo Superior de Investigaciones Científicas (CSIC), 28049 Madrid, Spain; mc.terradas@csic.es; 3 Departamento de Química en Ciencias Farmacéuticas, Facultad de Farmacia, Universidad Complutense de Madrid, Instituto de Investigación Sanitaria Hospital 12 de Octubre i+12, Plaza Ramón y Cajal s/n, 28040 Madrid, Spain; 4 CIBER de Bioingeniería, Biomateriales y Nanomedicina, CIBER-BBN, 28040 Madrid, Spain

**Keywords:** mesoporous bioactive glass, osteogenesis, angiogenesis, endothelial progenitor cells, pre-osteoblasts, macrophages

## Abstract

Due to their specific mesoporous structure and large surface area, mesoporous bioactive glasses (MBGs) possess both drug-delivery ability and effective ionic release to promote bone regeneration by stimulating osteogenesis and angiogenesis. Macrophages secrete mediators that can affect both processes, depending on their phenotype. In this work, the action of ion release from MBG-75S, with a molar composition of 75SiO_2_-20CaO-5P_2_O_5_, on osteogenesis and angiogenesis and the modulatory role of macrophages have been assessed in vitro with MC3T3-E1 pre-osteoblasts and endothelial progenitor cells (EPCs) in monoculture and in coculture with RAW 264.7 macrophages. Ca^2+^, phosphorous, and silicon ions released from MBG-75S were measured in the culture medium during both differentiation processes. Alkaline phosphatase activity and matrix mineralization were quantified as the key markers of osteogenic differentiation in MC3T3-E1 cells. The expression of CD31, CD34, VEGFR2, eNOS, and vWF was evaluated to characterize the EPC differentiation into mature endothelial cells. Other cellular parameters analyzed included the cell size and complexity, intracellular calcium, and intracellular content of the reactive oxygen species. The results obtained indicate that the ions released by MBG-75S promote osteogenesis and angiogenesis in vitro, evidencing a macrophage inhibitory role in these processes and demonstrating the high potential of MBG-75S for the preparation of implants for bone regeneration.

## 1. Introduction

Mesoporous bioactive glasses (MBGs) are bioceramics mainly designed for bone tissue repair because of their ability to mimic bone mineralization processes in contact with biological fluids, which allows them to bind to adjacent bone tissue [1]. Furthermore, the possibility of introducing drugs into the pores of these mesoporous materials, proved in pioneer work by Professor María Vallet-Regí 20 years ago, and the ability to tailor pore structure and textural properties at the nanoscale, results in a very precise control of the loading and release capabilities of different pharmaceuticals, such as anti-osteoporotic, antimicrobial, or antitumoral agents [2,3,4]. Since early studies by authors such as Vallet-Regí, Hench, and Zhao (back in the early 2000s), the field of MBGs for bone tissue repair has experienced a remarkable scientific explosion, with over 700 scientific publications to date (source: Scopus). Equally important is the action of the ions released by the MBGs, which presents the ability to stimulate key processes, such as osteogenesis and angiogenesis [5,6], and may also influence the immune response to these biomaterials [7].

Different strategies and experimental models can be used to evaluate, in vitro, the action of the ions released by MBGs in all these essential processes. The experimental approaches are focused on evaluating the specific responses of the cell types responsible for osteogenesis, angiogenesis, and the immune response to these biomaterials, involving the use of cultured bone cells, endothelial cells, and immune cells. Recently, cocultures of these cell types are being used, as they represent a more physiological experimental model in which different cells can communicate with each other as they would in vivo [8,9]. For the in vitro assessment of the response of mature osteoblasts to MBGs, human Saos-2 and MG-63 cells are excellent models as “osteoblast-like” cell lines, although with different sensitivities [10,11,12]. However, to study the effects of these biomaterials on the osteogenesis process, the MC3T3-E1 cell line is the model of choice. MC3T3-E1 pre-osteoblasts have a proliferation, differentiation, and mineralization capacity that is comparable to primary human pre-osteoblasts [13,14], being the most frequently employed model of in vitro osteogenesis [15,16].

Regarding the evaluation of the action of these biomaterials and their products on the process of angiogenesis, the use of endothelial progenitor cells (EPCs) directly involved in the formation of blood vessels, offers great advantages as an experimental model [16,17,18]. These cells have a high potential for tissue engineering applications and a highly proliferative antithrombogenic behavior; additionally, they can be easily obtained from peripheral blood [19,20,21,22].

Concerning the effects of any biomaterial on macrophages, it is important to note that these cells are directly involved in the innate immune response and can play both positive and negative roles in the biomaterial integration [23] due to the plasticity between the two phenotypes, characterized by the expression of specific cell-surface markers and the secretion of different cytokines [24]. Thus, the M1 pro-inflammatory macrophages are characterized by high expression of CD80, high-inducible nitric oxide synthase (iNOS) levels, and the release of pro-inflammatory cytokines [25,26]. The M2 reparative macrophages are involved in tissue repair, possess pro-angiogenic properties, and are characterized by the secretion of several anti-inflammatory cytokines and the expression of CD206, CD163, and MHC class II receptors [26,27,28]. The M2 macrophages can be divided into M2a, M2b, M2c, and M2d macrophages that are induced by different stimuli and produce specific molecules with different activities [29,30]. It is important to note that M2d macrophages are induced by agonists that target toll-like receptors (TLRs) through the adenosine receptor, followed by the secretion of both anti-inflammatory cytokines and vascular endothelial growth factors (VEGF) with pro-angiogenic properties [31,32]. In the biocompatibility studies of biomaterials, different parameters are generally analyzed to determine if the biomaterial in question induces polarization towards a pro-inflammatory M1 phenotype or towards a reparative M2 phenotype. These parameters include specific markers detected by flow cytometry and the secretion of cytokines, characteristics of each phenotype are quantified by ELISA techniques [9,24,25].

In this work, the actions of ion release from MBG-75S [10] with nominal molar composition 75SiO_2_-20CaO-5P_2_O_5_ on the differentiation of MC3T3-E1 pre-osteoblasts and endothelial progenitor cells (EPCs), in monoculture and in coculture with RAW 264.7 macrophages, have been evaluated in order to know the potential of this material and to promote osteogenesis and angiogenesis, as well as to assess the modulatory role of macrophages in these processes. The alkaline phosphatase activity (ALP) of MC3T3-E1 cells and the expression of specific EPC markers (CD31, CD34, VEGFR2, eNOS, and vWF) were quantified to characterize the differentiation of these two cell types into mature cells. Cell size and complexity, intracellular calcium, and intracellular content of the reactive oxygen species (ROS) were chosen as biocompatibility parameters. The evaluation of the action of ion release from MBG-75S on the intracellular ROS in the EPCs is especially important because it has been demonstrated that oxidative stress impairs the function of this cell type [21].

## 2. Materials and Methods

### 2.1. Synthesis and Characterization of MBG-75S

A mesoporous bioactive glass, MBG-75S of molar composition 75SiO_2_-20CaO-5P_2_O_5_, was prepared following the evaporation-induced self-assembly method [33] and using a non-ionic triblock copolymer (PEO)100-(PPO)65-(PEO)100 [PEO is poly(ethylene oxide) and PPO is poly(propylene oxide)], pluronic F127 (Sigma-Aldrich Corporation, St. Louis, MO, USA), as structure directing agent, as described in a previous work [10]. Briefly, 32 g of Pluronic F127 were dissolved in a solution of 15 mL of HCl 0.5 M and 485 mL of ethanol. Afterwards, 61.3 mL of tetraethylortosilane, Si(OC_2_H_5_)_4_, TEOS (Sigma-Aldrich Corporation, St. Louis, MO, USA), 6.28 mL of triethylphosphate, P(OC_2_H_5_)_3_, TEP (Sigma-Aldrich Corporation, St. Louis, MO, USA) and 17.6 mg of calcium nitrate tetrahydrate, Ca(NO_3_)_2_·4H_2_O (Sigma-Aldrich Corporation, St. Louis, MO, USA), were added at three hours intervals. The mixture was magnetically stirred for 24 h and then poured into Petri dishes to evaporate at 30 °C for 7 days. After this period, the material was removed from the dishes as transparent and homogeneous membranes, which were heated at 700 °C for 3 h in order to remove nitrates and organic compounds. Finally, the resulting powder was milled and sieved, collecting the grain-size fraction below 40 micrometers.

In order to study the mesoporous structure of MBG-75S, the resulting powder was analyzed by low-angle X-ray diffraction using a Philips X’Pert diffractometer (Cu Kα radiation, wavelength 1.5406 Å, Philips Analytical Company, Eindhoven, The Netherlands), between 0.5 and 6.5 2θ° angle and transmission electron microscopy (TEM) using a JEOL-1400 microscope operating at 300 kV (Cs 0.6 mm, resolution 1.7 Å, JEOL Ltd., Tokyo, Japan). Chemical composition was determined by energy-dispersive X-ray spectroscopy (EDX, JEOL Ltd., Tokyo, Japan) measurements (during TEM experiments) and X-ray fluorescence (XRF) spectroscopy, using a Philips PANalytical AXIOS spectrometer (Philips Electronics NV, Philips Analytical Company, Eindhoven, The Netherlands), with X-rays generated by the RhKα line at λ = 0.614 Å. Textural properties (surface area and porosity) were determined by nitrogen adsorption/desorption analysis using ASAP 2020 equipment (Micromeritics Instrument, Norcross, GA, USA). The MBG-75S was previously degassed under vacuum for 15 h, at 150 °C

### 2.2. Measurement of Ion Release from MBG-75S into the Culture Medium

The levels of soluble calcium, phosphates, and silica species in the culture medium were measured by inductively coupled plasma (ICP) spectroscopy, after soaking MBG-75S in Dulbecco’s Modified Eagle Medium (DMEM, Sigma Chemical Company, St. Louis, MO, USA) (1 mg/mL) for 3 and 7 days.

### 2.3. Culture and Differentiation of MC3T3-E1 Pre-Osteoblasts. Indirect Treatment with MBG-75S

The MC3T3-E1 pre-osteoblasts, provided by Dr. B.T. Pérez-Maceda (CIB, CSIC, Madrid, Spain), were seeded at a density of 2 × 10^4^ cells/mL in 6-well culture plates (Corning Inc., Corning, NY, USA) with 3 mL of α-Modified Eagle Medium (α-MEM, Sigma-Aldrich Corporation, St. Louis, MO, USA) with fetal bovine serum (FBS, Gibco BRL, Paisley, UK, 10% *v*/*v*), 1 mM L-glutamine (BioWhittaker Europe, Verviers, Belgium) and antibiotics (200 μg penicillin and 200 μg streptomycin per mL, BioWhittaker Europe, Verviers, Belgium). This medium was supplemented with 10 mM L-ascorbic acid and 50 μg/mL β-glycerolphosphate (Sigma-Aldrich Corporation, St. Louis, MO, USA) to promote cell differentiation. Cells were cultured for different times in a 5% CO_2_ incubator at 37 °C and were harvested with trypsin-EDTA (0.25%) to measure, by flow cytometry, the different cellular parameters indicated below. To analyze the effect of the ions released by MBG-75S on the in vitro differentiation process of MC3T3-E1 pre-osteoblasts, 1 mg/mL of MBG-75S was added on 6-well transwell inserts (0.4 µm pore size, Corning Inc., Corning, NY, USA), which were covered by the culture medium, allowing the ions to diffuse through the pores. The use of transwell inserts allows for the evaluation of only the actions of the released ions, in order to avoid the effects that could be generated by the physical presence of the material itself.

### 2.4. Cell Size and Complexity Analysis

Cell size and complexity were analyzed by detecting forward angle (FSC) and side angle (SSC) scatters, respectively, in a FACScalibur Becton Dickinson flow cytometer. To obtain statistically significant results, 10^4^ cells were analyzed in each sample.

### 2.5. Intracellular Reactive Oxygen Species (ROS) Content

Cell suspensions were incubated with 100 μM of 2′,7′-dichlorofluorescein diacetate (DCFH/DA, Serva, Heidelberg, Germany) for 30 min at 37 °C. DCFH/DA penetrates into the cells, where it is hydrolyzed by cytosolic esterases and produces DCFH, which is instantly oxidized by the intracellular reactive oxygen species (ROS) to the highly fluorescent DCF. The fluorescence intensity of DCF is directly dependent on the intracellular ROS content and was measured on a FACScalibur Becton Dickinson flow cytometer with a 530/30 filter, exciting the sample at 488 nm. To obtain statistically significant results, 10^4^ cells were analyzed in each sample.

### 2.6. Intracellular Calcium Content

Cell suspensions were incubated for 30 min with the Fluo4-AM probe (5 μM, Thermo Fisher Scientific, Madrid, Spain), which penetrates the cells and is then hydrolyzed by cytosolic esterases, releasing Fluo4 (with fluorescence directly proportional to the intracellular calcium content). The fluorescence of Fluo4 was measured on a FACScan Becton Dickinson flow cytometer with a 530/30 filter, exciting the sample at 488 nm. Finally, to verify the sensitivity of the assay, the ionophore A-23187 (5 μM, Sigma-Aldrich, St. Louis, MO, USA) was added to each sample. To obtain statistically significant results, 10^4^ cells were analyzed in each sample.

### 2.7. Alkaline Phosphatase Activity (ALP)

Alkaline phosphatase (ALP) is an enzyme produced by mature osteoblasts and is directly involved in matrix mineralization [34]. For this reason, ALP activity is widely used as a marker of differentiation of MC3T3-E1 pre-osteoblasts to the osteoblastic phenotype. To measure ALP activity, we used a commercial kit (SpinReact S.A., Barcelona, Spain) and the values obtained were referred to total protein content using the Bradford method and bovine serum albumin (BSA) as standard (SpinReact S.A., Barcelona, Spain).

### 2.8. Mineralization Assay

After differentiation of MC3T3-E1 pre-osteoblasts, the culture medium was removed, and the cells were treated with glutaraldehyde (10%) for 1 h. The cells were then stained with 40 mM alizarin red at pH 4.2 for 45 min and the stained extracellular deposits were dissolved with cetylpyridinium chloride (10% at pH 7), measuring the absorbance of the supernatants at 620 nm.

### 2.9. Obtention, Culture, and Characterization of Endothelial Progenitor Cells (EPCs). Indirect Treatment with MBG-75S

Endothelial progenitor cells (EPCs) were obtained, as previously described [35,36]. Briefly, porcine whole blood was diluted in phosphate buffered saline (PBS, 1:1) with 0.1% bovine serum albumin (BSA) and 0.6% sodium citrate. Mononuclear cells (MNC) were isolated using a density gradient prepared with Histopaque-1077 solution (Sigma-Aldrich Corporation, St. Louis, MO, USA) in AccuspinTM tubes (Sigma-Aldrich Corporation, St. Louis, MO, USA). After centrifugation at 800× *g* for 30 min, the MNC layer was collected and seeded in endothelial growth medium (EGM-2, Sigma-Aldrich Corporation, St. Louis, MO, USA) in F75 polystyrene culture flasks (Corning Inc., Corning, NY, USA) at a density of 2–3 × 10^5^ cells/cm^2^ under a CO_2_ (5%) atmosphere and at 37 °C. The culture medium was replaced at 96 h and then every 48 h until confluence.

To evaluate the differentiation of EPCs towards a mature endothelial phenotype after 23 and 30 days of culture in EGM-2, the expression of the following specific markers was assessed by flow cytometry: CD31 (transmembrane receptor, also termed platelet endothelial cell adhesion molecule-1, PECAM-1) [37], CD34 (cell-surface transmembrane glycoprotein, selectively expressed in the haematopoietic system on stem and progenitor cells) [38], eNOS (endothelial nitric oxide synthase, responsible for endothelial NO production) [39], vWF (von Willebrand factor, glycoprotein produced by endothelial cells, and megakaryocytes) [40], and VEGFR2 (VEGF receptor 2 (whose expression is restricted to endothelial cells), monocytes, and haematopoietic precursors) [41]. For immunostaining and identification of these markers by flow cytometry, the following antibodies were used with samples containing 10^6^ EPCs: anti-CD31 (TLD-3A12, ab64543, Abcam, UK), anti-CD34 (EP373Y, ab81289, Abcam, UK), anti-eNOS (M221, ab76198, Abcam, UK), anti-vWF (ab6994, Abcam, UK), and anti-VEGFR2 (ab2349, Abcam, UK). Two secondary antibodies were used conjugated with either DyLight 633 (IgG (H + L) goat anti-rabbit DyLight^®^ 633, Invitrogen, CA, USA) or Alexa 488 (IgG (H + L) Highly Cross-Adsorbed goat anti-mouse Alexa Fluor^®^ Plus 488, Invitrogen, CA, USA). All antibodies were prepared in 2% normal goat serum (NGS) and the final antibody concentrations were 10 µg/mL, unless specifically prescribed by the supplier. For intracellular markers (eNOS and vWF), cells were previously permeabilized with 0.25% saponin for 10 min at 4 °C before addition of the antibodies and 0.25% saponin was maintained for all subsequent immunolabelling steps. For the immunostaining, cells were previously incubated in 10% PBS/NGS for 10 min at room temperature to avoid non-specific binding and sedimented after this by centrifugation. The pellet was then treated with 300 µL of the corresponding primary antibody and incubated for 30 min at room temperature in the dark. Then, cells were washed by addition of 700 µL of 1% PBS/BSA, sedimented by centrifugation, and the pellet was incubated with 300 µL of the corresponding secondary antibody for 30 min at room temperature in the dark. Finally, cells were washed with 700 µL of 1% PBS/BSA for 10 min at room temperature, centrifuged, and resuspended in 300 µL of PBS. The EPC suspensions were then analyzed on a FACScalibur Becton Dickinson flow cytometer. Alexa Fluor 488 fluorescence was excited at 488 nm and measured at 519 nm. DyLight 633 fluorescence was excited at 638 nm and measured at 658 nm.

To analyze the effect of the ions released by MBG-75S on the in vitro differentiation process of EPCs, 1 mg/mL of MBG-75S was added on 6-well transwell inserts (0.4 µm pore size, Corning Inc., Corning, NY, USA), which is covered by the culture medium, allowing the ions to diffuse through the pores. As it has been indicated above in Section 2.3, the use of transwell inserts allows for the evaluation of only the action of the released ions, in order to avoid the effects that could be generated by the physical presence of the material itself.

### 2.10. Culture and Indirect Treatment of RAW 264.7 Macrophages with MBG-75S. Exposure of MC3T3-E1 Pre-Osteoblasts to Macrophage Conditioned Media

**Scheme 1:**
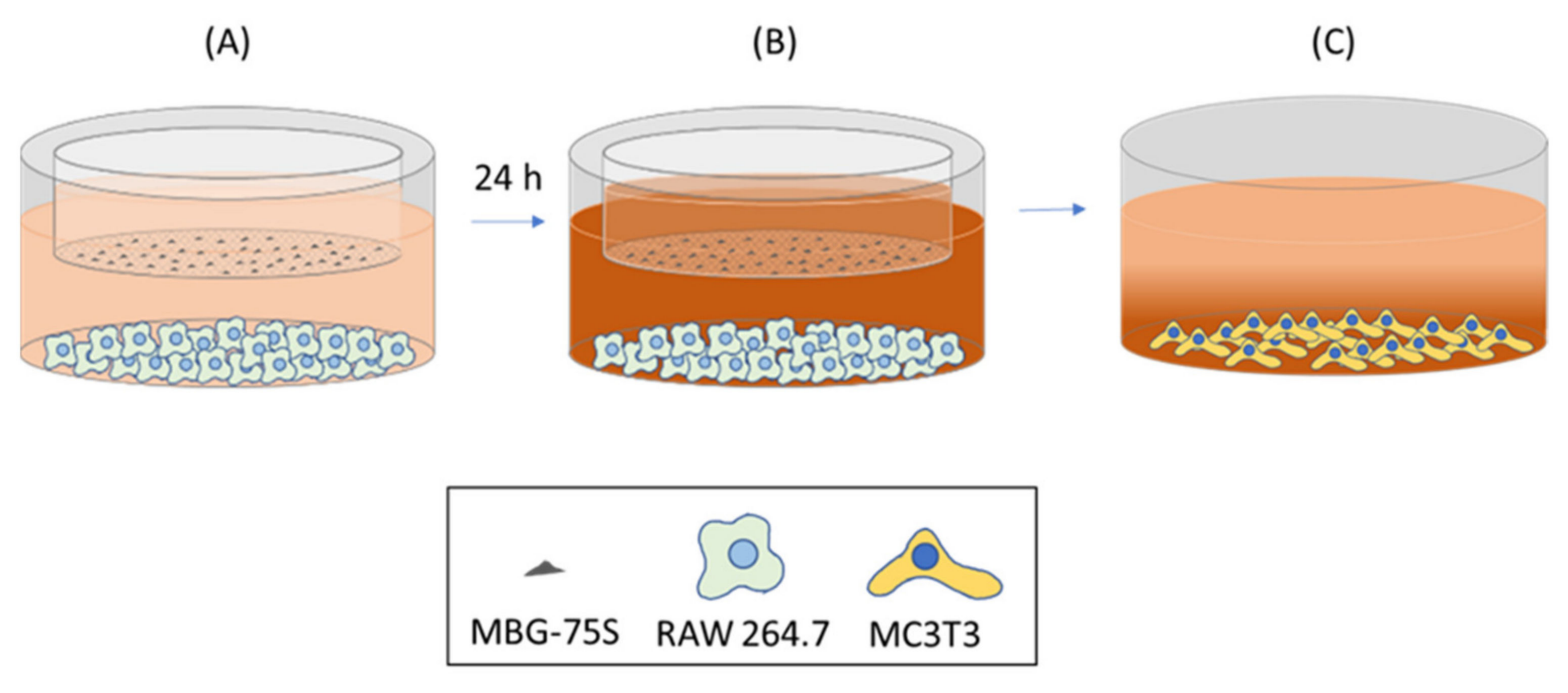
Preparation of conditioned media of RAW 264.7 macrophages and treatment of MC3T3-E1 pre-osteoblasts. RAW 264.7 macrophages were indirectly treated with 1 mg/mL MBG-75S, deposited on 6-well transwell inserts, which is covered by DMEM (**A**), after 24 h of culture (**B**), and the media from these macrophage cultures were collected and diluted 1:1 with α-MEM for the subsequent treatment of MC3T3 -E1 pre-osteoblasts for 10 days (**C**).

RAW 264.7 macrophages (American Type Culture Collection, ATCC, Manassas, VA, USA) were seeded in 6-well culture plates (Corning Inc., Corning, NY, USA), at a density of 1.5 × 10^5^ cells/mL, in 3 mL of Dulbecco’s modified eagle medium (DMEM, Sigma-Aldrich Corporation, St. Louis, MO, USA) supplemented with 10% fetal bovine serum (FBS, Gibco, BRL), 1 mM L-glutamine (BioWhittaker Europe, Verviers, Belgium), penicillin (200 µg/mL, BioWhittaker Europe, Verviers, Belgium), and streptomycin (200 µg/mL, BioWhittaker Europe) at 37 °C under a CO_2_ (5%) atmosphere. To analyze the effects of the ions released by MBG-75S, concerning the possible regulatory role of macrophages on in vitro osteogenesis, RAW 264.7 cells were cultured in the absence (control macrophages) or in the presence of 1 mg/mL of MBG-75S deposited on 6-well transwell inserts (0.4 µm pore size, Corning Inc., Corning, NY, USA), which is covered by the culture medium. After 24 h of culture, the media from these macrophage cultures were collected and diluted 1:1 with α-MEM for subsequent treatment of MC3T3-E1 pre-osteoblasts (10^4^ cells/mL) for 10 days, as it is indicated in Scheme 1. ALP activity of MC3T3 -E1 cells was then assessed as specific marker of in vitro osteogenesis, as described in Section 2.7.

### 2.11. Coculture of MC3T3-E1 Pre-Osteoblasts and RAW 264.7 Macrophages. Indirect Treatment with MBG-75S and Measurement of ALP in MC3T3-E1 Cells

**Scheme 2:**
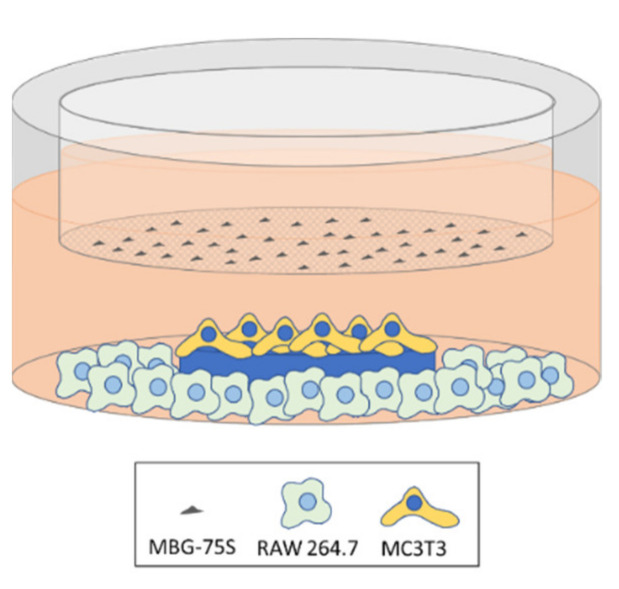
Coculture of MC3T3-E1 pre-osteoblasts with RAW 264.7 macrophages and indirect treatment with 1 mg/mL of MBG-75S deposited on transwell inserts.

10^4^ MC3T3-E1 pre-osteoblasts were seeded on glass coverslips introduced into 6-well culture plates (Corning Inc., Corning, NY, USA) and, simultaneously, 10^4^ RAW 264.7 macrophages were seeded at the bottom of each well, around each coverslip, as shown in Scheme 2, adding 3 mL of culture medium composed of α-MEM and DMEM 1:1. These cocultures were performed during 10 days in the presence of 1 mg/mL of MBG-75S deposited on 6-well transwell inserts (0.4 µm pore size, Corning Inc., Corning, NY, USA) at 37 °C under a CO_2_ (5%) atmosphere. After coculturing, MC3T3-E1 cells were washed with PBS, harvested using 0.25% trypsin-EDTA solution and ALP activity was measured, as described above in Section 2.7.

### 2.12. Coculture of EPCs and RAW 264.7 Macrophages. Indirect Treatment with MBG-75S and Measurement of VEGFR2 Expression in EPCs and CD206 Expression in RAW 264.7 Macrophages

**Scheme 3:**
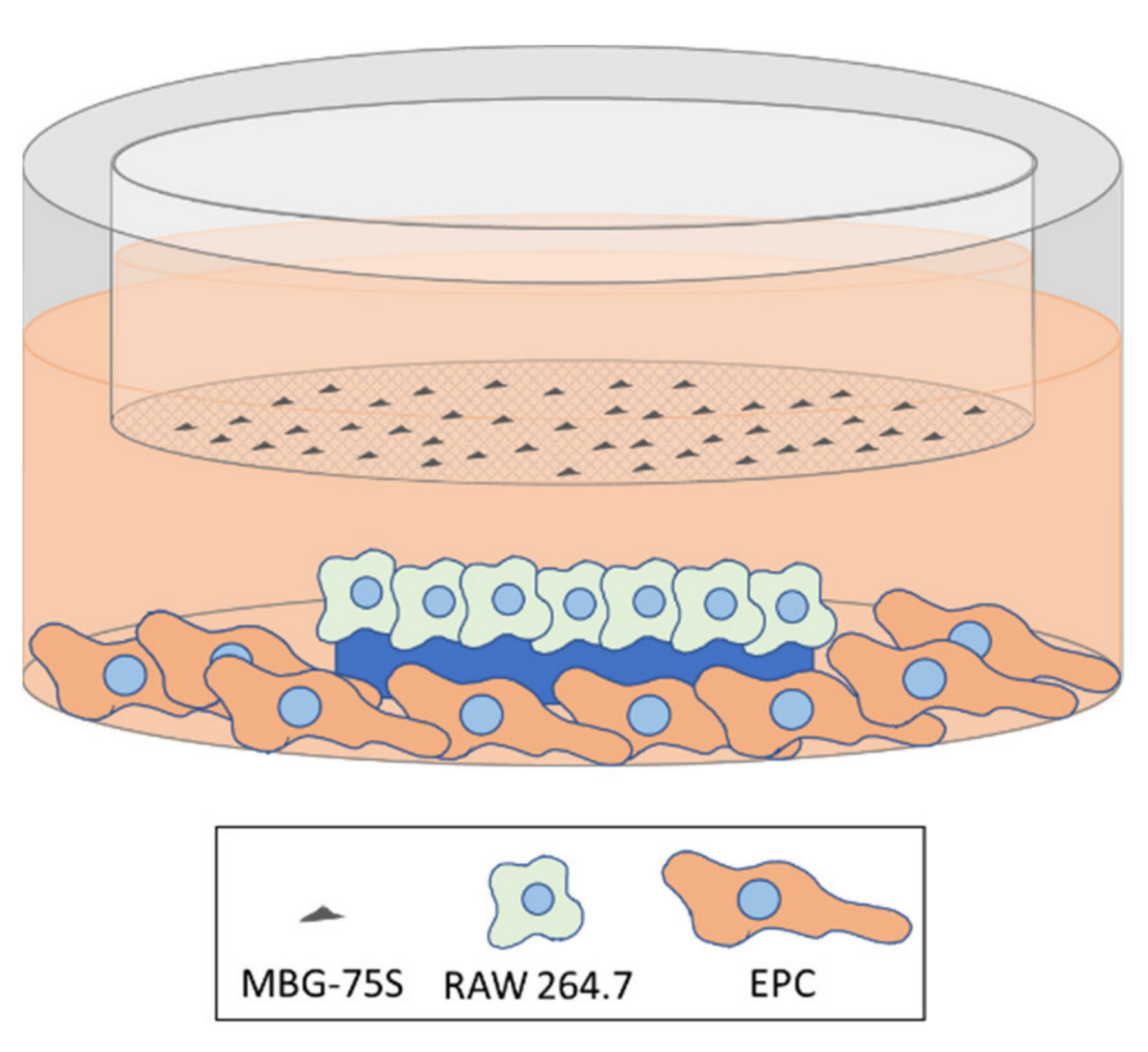
Coculture of EPCs with RAW 264.7 macrophages and indirect treatment with 1 mg/mL of MBG-75S deposited on transwell inserts.

1.5 × 10^4^ RAW 264.7 macrophages were seeded on glass coverslips introduced into 6-well culture plates (Corning Inc., Corning, NY, USA) and, simultaneously, 1.5 × 10^4^ EPCs were seeded at the bottom of each well, around each coverslip, as shown in Scheme 3, adding 3 mL of culture medium composed of EGM-2 and DMEM 1:1. These cocultures were performed during 7 days in the presence of 1 mg/mL of MBG-75S deposited on 6-well transwell inserts (0.4 µm pore size, Corning Inc., Corning, NY, USA) at 37 °C under a CO_2_ (5%) atmosphere. After coculturing, EPCs and macrophages were washed with PBS, harvested using 0.25% trypsin-EDTA solution and cell scrapers, respectively, centrifuged at 310× *g* for 10 min, and resuspended in PBS for the analysis of the expression of VEGFR2 in EPCs and CD206 in RAW 264.7 macrophages by flow cytometry. VEGFR2 was detected as a specific marker of EPCs differentiation into mature endothelium, as described above in Section 2.9. On the other hand, CD206 was detected as a specific marker of M2 macrophage phenotype [24] by flow cytometry. With this objective, after detachment, macrophages were centrifuged and incubated in 45 µL of staining buffer (PBS, 2.5% FBS Gibco, BRL, and 0.1% sodium azide, Sigma-Aldrich Corporation, St. Louis, MO, USA) with 5 µL of normal mouse serum inactivated for 15 min at 4 °C to block the Fc receptors, before adding the primary antibody, and to prevent nonspecific binding. Then, cells were incubated with FITC anti-mouse CD206 (15 µg/mL, BioLegend, San Diego, CA, USA) for 30 min in the dark. Labeled cells were analyzed using a FACSCalibur flow cytometer. FITC fluorescence was excited at 488 nm and measured with a 530/30 band pass filter.

The effect of the ions released by MBG-75S on the expression of VEGFR2 and CD206 in EPC/macrophage cocultures were compared with the expression of these specific markers when EPCs were cocultured with M2d angiogenic macrophages, obtained from RAW 264.7 macrophages treated with 5′-Nethyl-carboxamido-adenosine (NECA, 1 µM, MedChemExpress, Monmouth Junction, NJ, USA)/*E. coli* LPS (100 ng/mL, Sigma-Aldrich Corporation, St. Louis, MO, USA) [32].

### 2.13. Statistics

Results were expressed as the means of three identical experiments, with their corresponding standard deviations, analyzed with the 22th version of Statistical Package for the Social Sciences (SPSS). Statistical comparisons were carried out by one-way analysis of variance (ANOVA) and Scheffé and Games-Howell tests were used for post-hoc analysis of differences between study groups, considering *p* < 0.05 as statistically significant.

## 3. Results and Discussion

### 3.1. Characterization of MBG-75S

**Figure 1:**
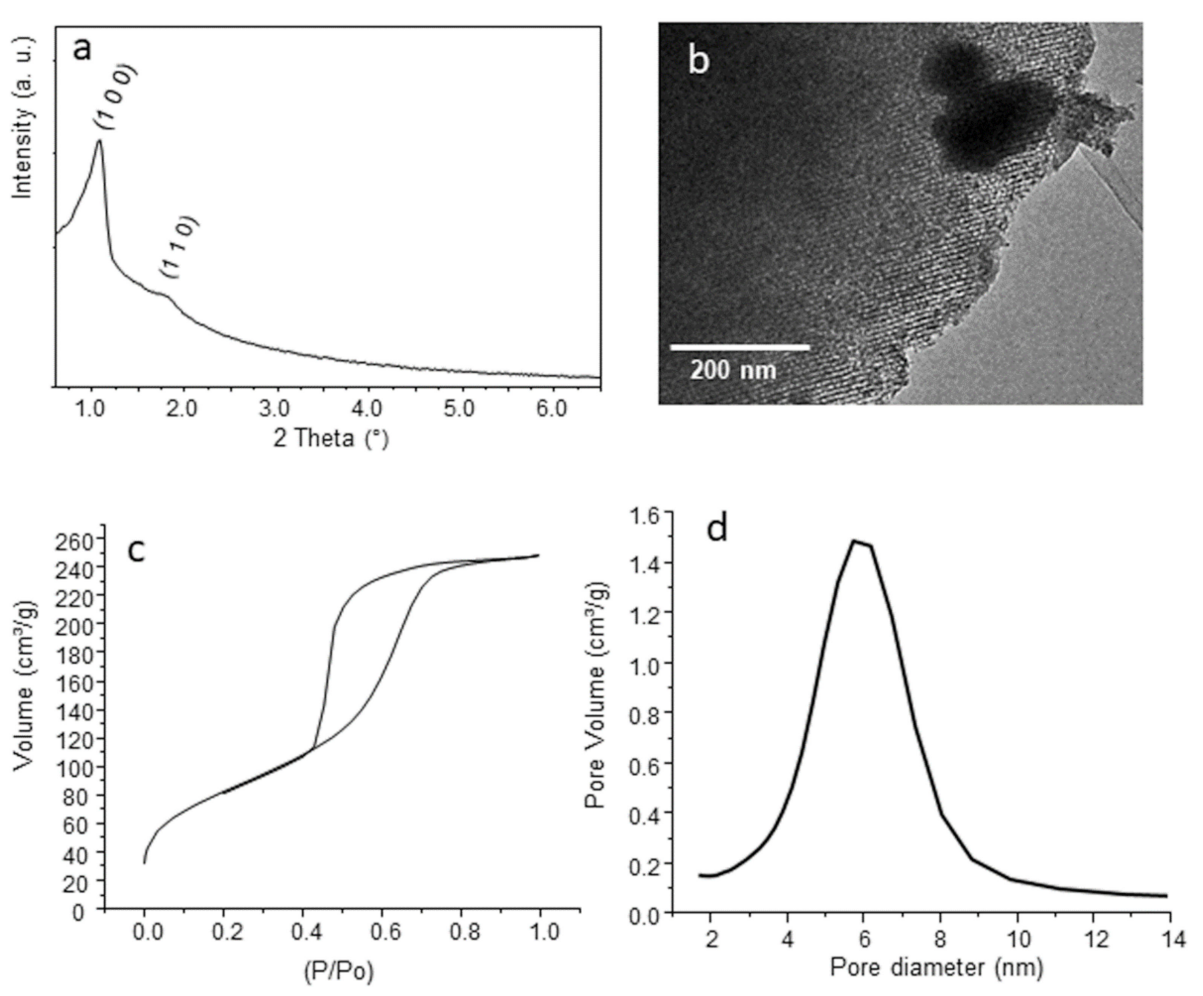
Low-angle X-ray diffraction pattern obtained from MBG-75S. The numbers in brackets indicate the Miller indexes for a p6mm planar spatial group (**a**). TEM image and EDX spectrum (inset) obtained from MBG-75S showing an ordered channel like mesoporous structure compatible with the 2D-hexagonal p6mm group (**b**). Nitrogen adsorption/desorption isotherm (**c**). Pore size distribution (**d**).

The low-angle X-ray diffraction pattern collected for MBG-75S (Figure 1a) shows two diffraction maxima at 1.05 and 1.75 2θ° that can be assigned to (1 0 0) and (1 1 0) reflections, respectively, of a p6mm 2D-hexagonal planar group [42,43]. The ordered mesoporous structure was confirmed by TEM (Figure 1b) that shows a highly ordered channel structure characteristic of 2D-hexagonal p6m planar group. EDX spectra collected during TEM observation indicated a molar composition of 72.8SiO_2_-24.7CaO-2.5P_2_O_5_ (Figure 1b inset). Besides, XRF spectroscopy pointed out a molar composition (Table 1) that is consistent with EDX results and very similar to the nominal composition for MBG-75S.

Nitrogen adsorption/desorption analysis (Figure 1c) showed isotherms corresponding to mesoporous materials with a H1 hysteresis loop, indicating the presence of cylindrical shaped pores. The analysis confirmed the highly mesoporous structure of MBG-75S, showing values of surface area (S_BET_) and pore volume 305.5 m^2^·g^−1^ and 0.46 cm^3^·g^−1^, respectively. The pore size distribution (Figure 1d) shows as single modal distribution centered in 5.7 nm. These results are in agreement with those observed by XRD and TEM and confirm the highly ordered mesoporous structure of MBG-75S.

### 3.2. Ion Release from MBG-75S into the Culture Medium

**Figure 2:**
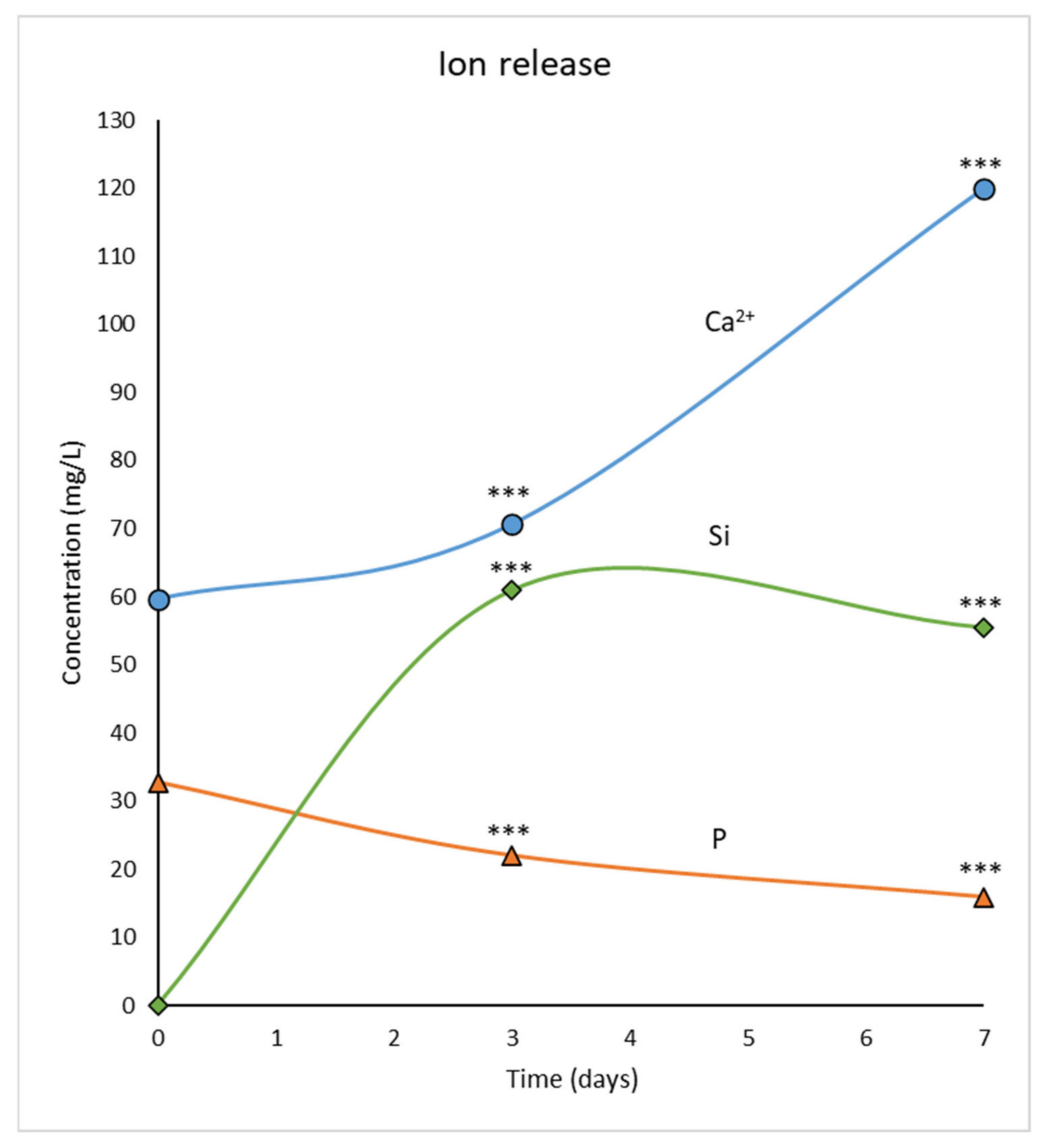
Ca^2+^, silicon, and phosphorous levels measured into the culture medium after soaking MBG-75S (1 mg/mL) in Dulbecco´s Modified Eagles Medium (DMEM) for 3 and 7 days. Statistical significance: *** *p* < 0.005.

To investigate the actions of ion release from MBG-75S, with the molar composition 75SiO_2_-20CaO-5P_2_O_5_, on the differentiation of osteoprogenitor and endothelial progenitor cells, the levels of Ca^2+^, silicon, and phosphorous were previously measured, after soaking MBG-75S (1 mg/mL) in Dulbecco's Modified Eagles Medium (DMEM) for 3 and 7 days. Significant increases (*p* < 0.005) of Ca^2+^ and Si levels were observed after 3 and 7 days, accompanied by a significant decrease (*p* < 0.005) in phosphorus concentration at these times (Figure 2). These results are consistent with earlier data from previous studies on the effects of this material on osteoblasts, osteoclasts, and macrophages [10].

### 3.3. Action of Ion Release from MBG-75S on Cell Size, Cell Complexity, Intracellular Reactive Oxygen Species, and Cytosolic Calcium of MC3T3-E1 Pre-Osteoblasts and Endothelial Progenitor Cells

**Figure 3:**
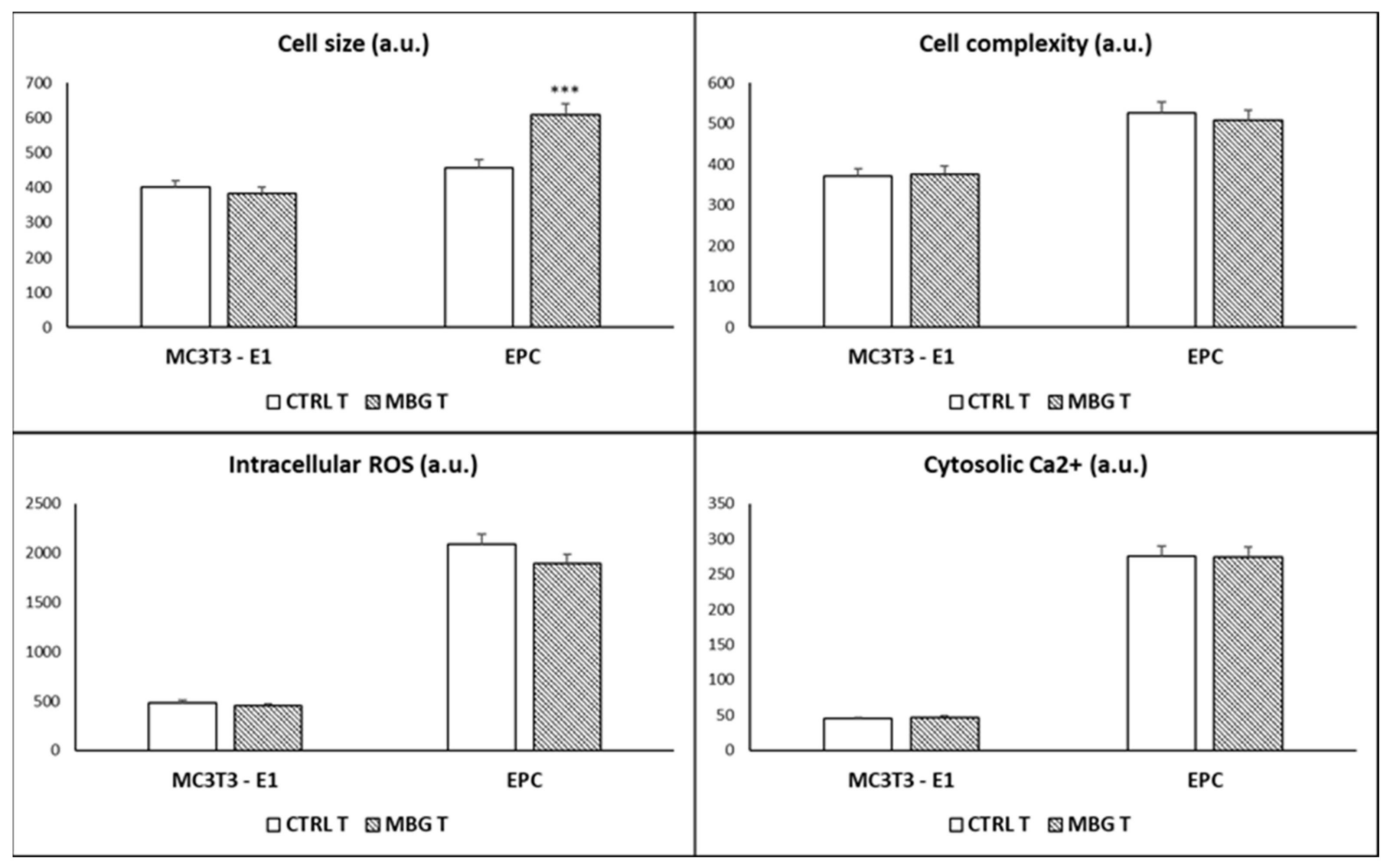
Action of ion release from MBG-75S on cell size, cell complexity, intracellular reactive oxygen species, and cytosolic calcium of MC3T3-E1 pre-osteoblasts and endothelial progenitor cells (EPCs) after 24 h. During cell culture, 1 mg/mL of MBG-75S was deposited on transwell inserts (MBG T), allowing the ions to diffuse through the pores in order to evaluate only the action of the released ions on each cell type. Control cells were cultured in the presence of transwell inserts without material (CTRL T) in parallel. Statistical significance: *** *p* < 0.005.

The early action of ions released by MBG-75S, on different the cell parameters of MC3T3-E1 pre-osteoblasts and endothelial progenitor cells (EPCs), was investigated before analyzing their effects on the differentiation of these two types of progenitor cells. The parameters chosen were cell size and complexity, intracellular content of the reactive oxygen species (ROS), and cytosolic Ca^2+^. As indicators of cell size and complexity, light scattering properties were examined by flow cytometry by measuring forward scattering (FSC) and side scattering (SSC), respectively. These characteristics depend, in part, on the volume of the cell, as well as the state of its plasma membrane, cytoplasm, mitochondria, lysosomes, and pinocytic vesicles [44]. As it can be observed in the upper right panel of Figure 3, the presence of 1 mg/mL of MBG-75S, deposited on transwell inserts (MBG T) allowing the ions to diffuse through the pores, did not produce significant changes in the complexity of these two cell types compared to the values observed in control cells cultured in the presence of transwell inserts without material (CTRL T). However, a significant increase in the size of the EPCs was produced by the ions released from MBG-75S (upper left panel of Figure 3). Preliminary work with the fibroblasts treated directly with non-calcined mesoporous matrices evidenced dose-dependent increases in fibroblast size, internal complexity, and intracellular calcium content, probably due to residual ethanol and the surface silanol groups [45]. However, significant dose-dependent decreases of osteoblast size and complexity, induced by direct treatment with powdered MBG-75S, were observed in another previous study, which could be due to the loss of cell anchorage produced by the presence of powdered material in the culture medium [10]. As it has been indicated above, the use of transwell inserts in the present work allows for the evaluation of the action of the released ions, avoiding the effects that could be generated by the physical presence of the material itself. However, the presence of alkali-earth metal cations, such as Ca^2+^, in silica-based glasses favors the silica network degradation as colloidal silicate fragments [46]. The biological importance of a fast initial dissolution of silicate fragments was quantitatively confirmed by Arcos et al. [47], demonstrating the relationship between the glass bioactivity with a low activation energy for silica release. As a hypothesis, the colloidal silica fragments could pass through the transwell membranes and be internalized by endothelial cells, thus resulting in a cell size increase, as described for most of mammalian adherent cells after nanoparticles uptake [48].

On the other hand, the ions released by MBG-75S did not induce significant changes in intracellular ROS content and cytosolic Ca^2+^, thus demonstrating that the soluble products of this material do not induce oxidative stress and do not alter the regulatory mechanisms of the intracellular calcium levels in both types of progenitor cells (lower left and right panels of the Figure 3). It is important to highlight that the evaluation of the action of ion release from MBG-75S on intracellular ROS in EPCs is especially important because it has been demonstrated that oxidative stress impairs the function of this cell type, which is involved in maintaining the integrity and function of the cardiovascular system [21].

### 3.4. Action of Ion Release from MBG-75S on Differentiation of MC3T3-E1 Pre-Osteoblasts. Modulating Role of Macrophages

**Figure 4:**
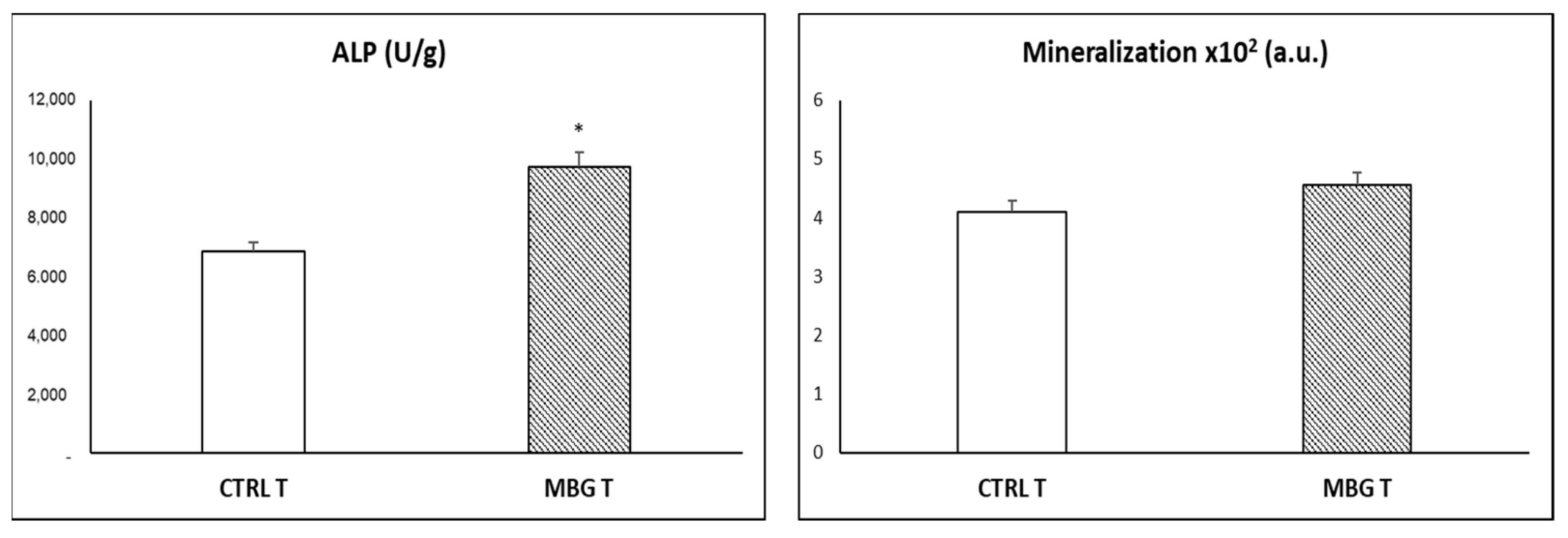
Action of ion release from MBG-75S on differentiation of MC3T3-E1 pre-osteoblasts. Alkaline phosphatase activity (ALP) and mineralization were evaluated as specific markers of in vitro osteogenesis after 10 days of culture in differentiation medium. During cell culture, 1 mg/mL of MBG-75S was deposited on transwell inserts (MBG T), allowing the ions to diffuse through the pores, in order to evaluate only the action of the released ions. Control cells were cultured in the presence of transwell inserts without material (CTRL T) in parallel. Statistical significance: * *p* < 0.05.

During the differentiation of MC3T3-E1 pre-osteoblasts, three stages can be identified: (a) early phase characterized by cell proliferation (but without expression of differentiation markers such as alkaline phosphatase (ALP) or mineral deposits), (b) intermediate phase with high expression of ALP and matrix maturation, and (c) final phase characterized by the presence of mineral deposits produced by ALP activity [49,50]. Figure 4 shows the action of ion release from MBG-75S on the differentiation of MC3T3-E1 pre-osteoblasts, assessed through the measure of ALP and mineralization as specific markers of in vitro osteogenesis. As it can be observed in the left panel of Figure 4, when 1 mg/mL of MBG-75S was deposited on transwell inserts (MBG T), to allow the ions to diffuse through the pores, a significant increase of ALP activity was observed after 10 days, in comparison to control cells cultured in the presence of transwell inserts without material (CTRL T). These results indicate the positive in vitro effect of ion release from MBG-75S on osteogenesis, in agreement with the previous results, obtained by us, with scaffolds prepared with this MBG and ε-polycaprolactone implanted in osteoporotic sheep [51], and by other authors, with mesoporous silica nanospheres, which stimulate osteogenesis by Si ion release [52]. Regarding the mineralization analysis, a slight increase of this parameter was also induced by the presence of MBG-75S deposited on the transwell inserts, but this effect was not statistically significant (right panel Figure 4). This slight effect on mineralization was probably due to the need of longer differentiation times, as well as the lower precision and sensitivity of this assay, in comparison with ALP measurement, as we have previously observed in other studies with mesoporous nanospheres and MC3T3-E1 pre-osteoblasts [53]. Several authors have demonstrated the pro-osteogenic effects of MBGs caused by to the release of calcium and silicon ions [54], and recent research has shown the influence of these kind of biomaterials on pulp cells behavior in vitro, increasing both ALP activity and mineralization in this cell type [55].

**Figure 5:**
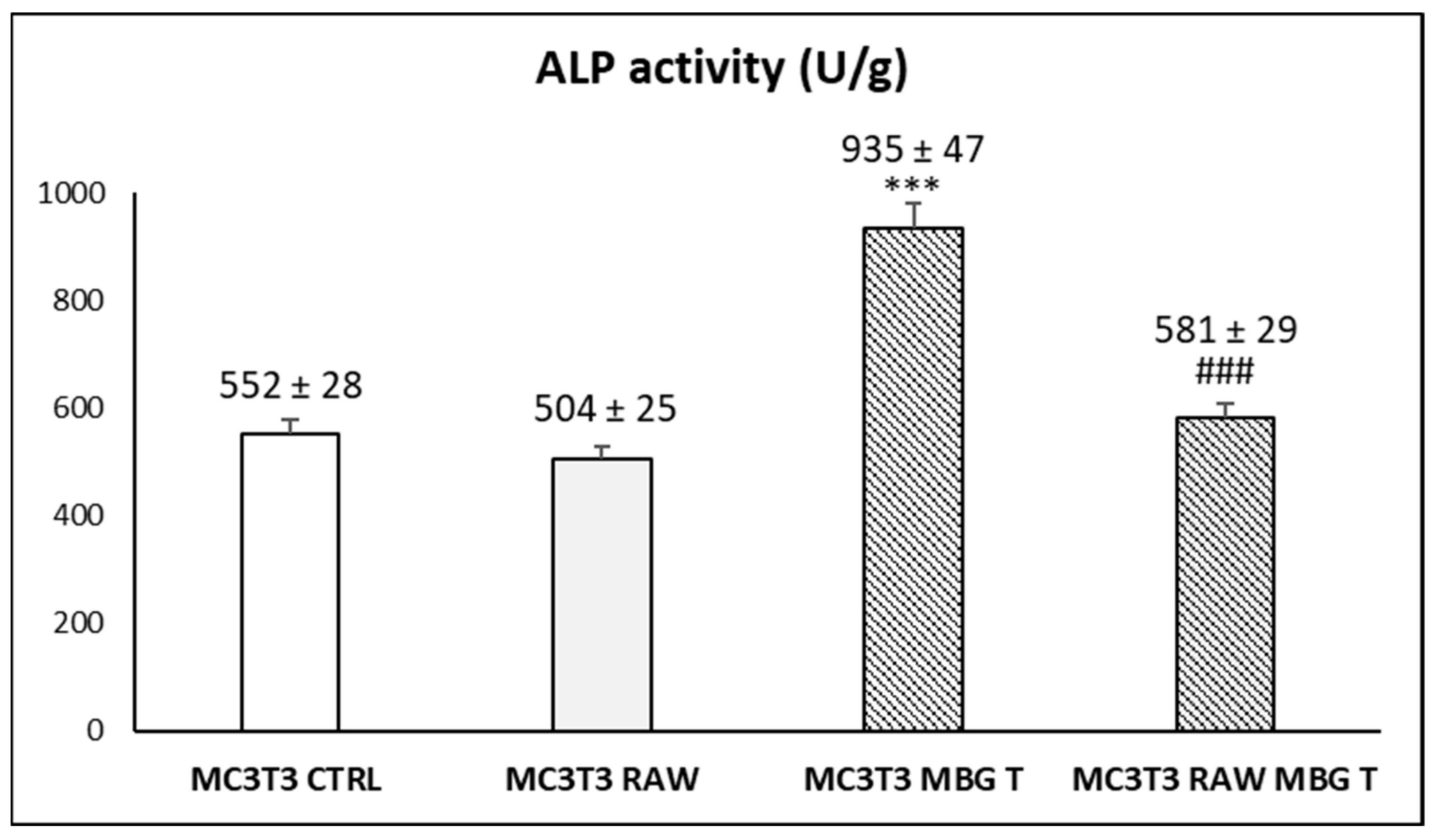
Modulating role of RAW 264.7 macrophages on the action of ion release from MBG-75S on differentiation of MC3T3-E1 pre-osteoblasts. Macrophages cultured in DMEM were indirectly treated with 1 mg/mL MBG-75S deposited on transwell inserts for 24 h, and the supernatants from these cultures were collected and diluted 1:1 with α-MEM. Control RAW 264.7 macrophages were cultured in parallel without material. Subsequently, MC3T3-E1 pre-osteoblasts were treated for 10 days with these conditioned media. Alkaline phosphatase activity (ALP) was evaluated as a specific marker of in vitro osteogenesis after 10 days of culture in the following conditions: MC3T3 CTRL = control pre-osteoblasts cultured in the absence of material, MC3T3 RAW = pre-osteoblasts cultured with medium of control RAW 264.7 macrophages in the absence of material, MC3T3 MBG T = pre-osteoblasts cultured in the presence of 1 mg/mL of MBG-75S deposited on transwell inserts, and MC3T3 RAW MBG T = pre-osteoblasts cultured with medium of RAW 264.7 macrophages previously treated with 1 mg/mL of MBG-75S deposited on transwell inserts. Statistical significance: *** *p* < 0.005 (comparison with MC3T3 CTRL); ### *p* < 0.005 (comparison with MC3T3 MBG T).

Very recent studies demonstrate the relationship between the immune response and bone regeneration, highlighting the crucial role of macrophages in the osteogenesis stimulated by biomaterials [56,57]. Macrophages, responsible for innate immunity, regulate the possible inflammatory reaction induced by the biomaterial, but they are also involved in tissue repair, depending on the polarization of macrophages towards the proinflammatory phenotype M1 or towards the reparative phenotype M2, respectively [23,24,25,26,27,28]. In this context, we wanted to assess the modulating role of RAW 264.7 macrophages, regarding the action of ion release from MBG-75S on the differentiation of the MC3T3-E1 pre-osteoblasts. With this objective, macrophages were cultured in the absence (control macrophages) or in the presence of 1 mg/mL of MBG-75S deposited on transwell inserts for 24 h. Then, the media from these macrophage cultures were collected and diluted 1:1 with α-MEM for the subsequent treatment of MC3T3-E1 pre-osteoblasts. Figure 5 shows the ALP activity of MC3T3-E1 after 10 days of differentiation, under the following conditions: MC3T3 CTRL = control pre-osteoblasts cultured in the absence of material, MC3T3 RAW = pre-osteoblasts cultured with medium of control RAW 264.7 macrophages in the absence of material, MC3T3 MBG T = pre-osteoblasts cultured in the presence of 1 mg/mL of MBG-75S deposited on transwell inserts, and MC3T3 RAW MBG T = pre-osteoblasts cultured with medium of RAW 264.7 macrophages previously treated with 1 mg/mL of MBG-75S deposited on transwell inserts.

Figure 5 highlights the positive effect on pre-osteoblast differentiation, induced by the release of ions from the 1 mg/mL of MBG-75S deposited on transwell inserts (MC3T3 MBG T), compared to control cells cultured in the absence of material (MC3T3 CTRL). On the contrary, the treatment of MC3T3-E1 preosteoblasts for 10 days, with a medium of control RAW 264.7 macrophages in the absence of material (MC3T3 RAW), induced a slight (but non-significant) decrease, compared to control pre-osteoblasts (MC3T3 CTRL). When the pre-osteoblasts were cultured with a conditioned medium of RAW 264.7 macrophages, previously treated with 1 mg/mL of MBG-75S deposited on transwell inserts (MC3T3 RAW MBG T), both opposite effects were present (the stimulation produced by the ions released by the MBG-75S material and the inhibition due to the mediators secreted by the macrophages). Thus, in the MC3T3 RAW MBG T condition, ALP values were higher than those obtained with control macrophage media (MC3T3 RAW), although this difference was not statistically significant. The comparison between the ALP values of pre-osteoblasts cultured in the presence MBG-75S deposited on transwell inserts (MC3T3 MBG T) and pre-osteoblasts cultured with a medium of RAW 264.7 macrophages previously treated with MBG-75S deposited on transwell inserts (MC3T3 RAW MBG T), evidences a pronounced and significant difference. This difference indicates that the ions released by MBG-75S have a positive effect if the material is in indirect contact with pre-osteoblasts but have no effect if the material was only in previous indirect contact with macrophages.

**Figure 6:**
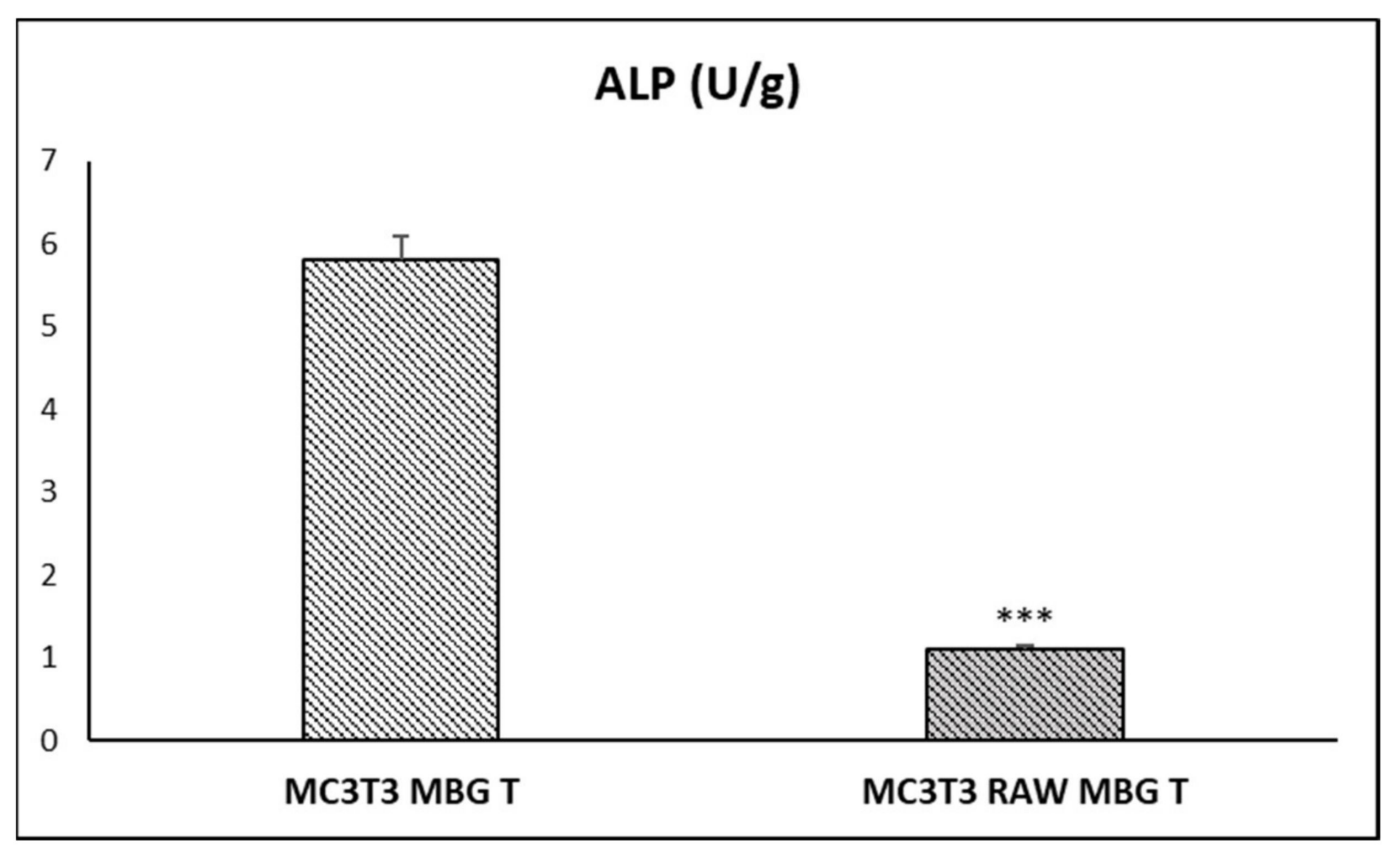
Comparison of the action of ion release from MBG-75S on differentiation of MC3T3-E1 pre-osteoblasts in monoculture (MC3T3 MBG T) and in coculture with RAW 264.7 macrophages (MC3T3 RAW MBG T). For the coculture, MC3T3-E1 pre-osteoblasts were seeded on glass coverslips introduced into 6-well culture plates and RAW 264.7 macrophages were seeded around each coverslip. Additionally, 1 mg/mL of MBG-75S was deposited on transwell inserts, allowing the ions to diffuse through the pores in both monocultures and cocultures. Alkaline phosphatase activity (ALP) was evaluated as a specific marker of in vitro osteogenesis after 10 days of culture. Statistical significance: *** *p* < 0.005.

To clarify on the possible inhibitory role of macrophages, we carried out the coculture of MC3T3-E1 pre-osteoblasts with RAW 264.7 macrophages and compared the action of ion release from MBG-75S on the differentiation of MC3T3-E1 pre-osteoblasts in monoculture and in coculture with these macrophages, as described in Section 2.11. The experimental coculture models are closer to the physiological conditions because different cell types can communicate with each other [8,9]. As it can be observed in Figure 6, ALP values obtained in MC3T3-E1 cells, after contact with the ions released by MBG-75S, were much lower when these cells were cocultured with macrophages (MC3T3 RAW MBG T) than they were in pre-osteoblast monocultures (MC3T3 MBG T). This pronounced, and statistically significant, difference suggests that macrophage-secreted mediators could inhibit the positive effect of the ions released by MBG-75S on the in vitro osteogenesis.

### 3.5. Differentiation and Phenotypic Characterization of EPCs

**Figure 7:**
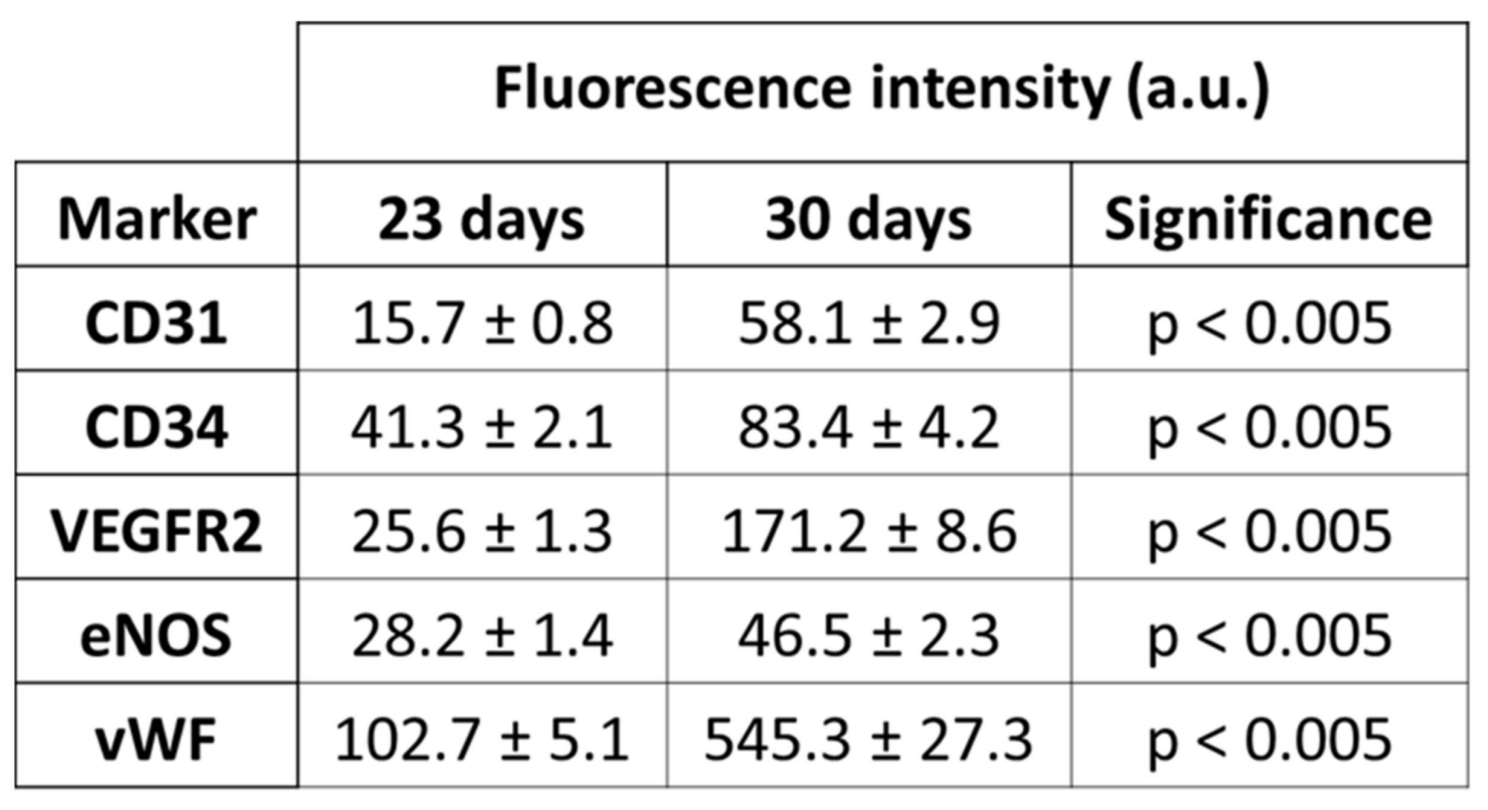
Phenotypic characterization of EPCs. The expression of CD31, CD34, VEGFR2, eNOS, and vWF each were evaluated by immunofluorescence labeling and flow cytometry in EPCs, as endothelial phenotype markers, after 23 and 30 days of differentiation.

The use of endothelial progenitor cells (EPCs), which are directly involved in blood vessel formation, offers great advantages as an experimental model for the evaluation of possible angiogenic effects of biomaterials [16,17,18] and has a high potential for tissue engineering applications [19,20,21,22]. In this work, EPCs were isolated from porcine blood and characterized by the expression of the endothelial phenotype markers CD31, CD34, VEGFR2, eNOS, and vWF after 23 and 30 days of differentiation, as described in Section 2.9. As it can be observed in Figure 7, the expression of all these markers significantly increased in a time-dependent manner, indicating the correct differentiation towards a mature endothelial phenotype. More pronounced increases were observed in VEGFR2 and vWF, in comparison to CD31, CD34, and eNOS. For this reason, and taking into account that the expression of VEGFR2 is directly related to angiogenesis, we chose VEGFR2 as a reference marker to evaluate the potential angiogenic effect of ion release from MBG-75S.

### 3.6. Action of Ion Release from MBG-75S on VEGFR2 Expression in EPCs

It is well known that VEGF acts on endothelial cells inducing angiogenesis [41] and regulating vascular permeability [58] by activating the VEGFR1 and VEGFR2 receptors, with VEGFR2 being the major signal transducer for angiogenesis through the PLCϒ–PKC–MAPK pathway [59]. The VEGF also promotes bone repair by stimulating angiogenesis, maturation of osteoblasts, ossification, and bone turnover [60]. Since osteogenesis is coupled to vascularization during bone growth [61], many current research efforts are focused on the development of angiogenic biomaterials to promote vascularization optimizing bone regeneration [16,18,62,63].

**Figure 8:**
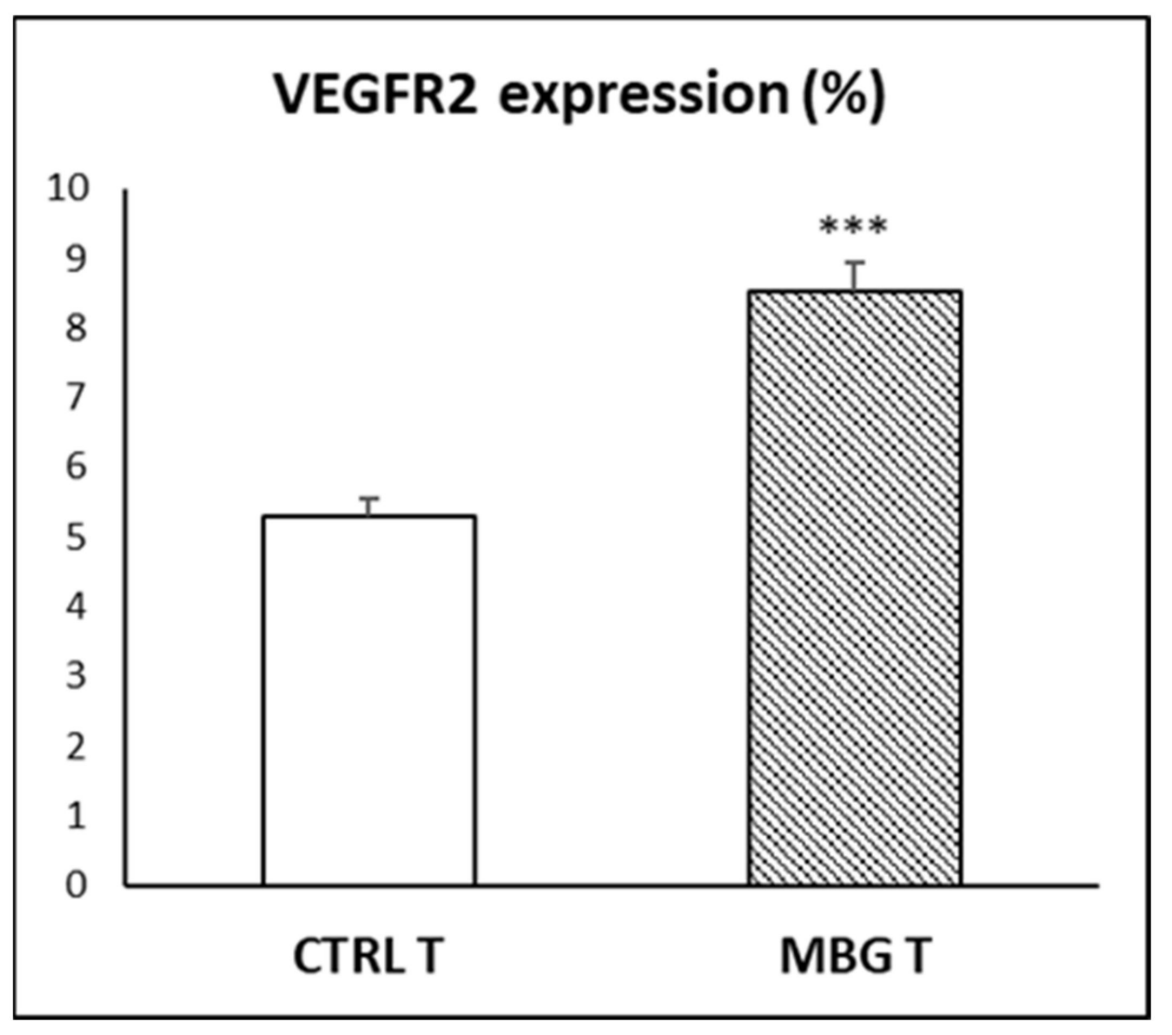
Action of ion release from MBG-75S on VEGFR2 expression in endothelial progenitor cells (EPCs) after 7 days of culture in differentiation medium. During cell culture, 1 mg/mL of MBG-75S was deposited on transwell inserts (MBG T), allowing the ions to diffuse through the pores, in order to evaluate only the action of the released ions. Control cells were cultured in the presence of transwell inserts without material (CTRL T) in parallel. Statistical significance: *** *p* < 0.005.

In this work, the action of ion release from MBG-75S on the differentiation process of EPCs was evaluated by quantifying the VEGFR2 expression as a specific marker of endothelial differentiation, directly related to angiogenesis. As it can be observed in Figure 8, the ion release from MBG-75S induced a significant and pronounced increase in the percentage of VEGFR2^+^ EPCs after 7 days of treatment (MBG T), compared to control cells (CTRL T). These results evidence the ability of the ions released from MBG-75S to stimulate VEGFR2 expression in EPCs and highlights their potential action on angiogenesis. The present in vitro study indicates that the incorporation of MBG-75S into bone tissue engineering scaffolds can be highly beneficial, due to its angiogenic potential (due mainly to the action of the ions released on endothelial progenitor cells). The obtained results would explain the effects recently observed by our group, in vivo, in an osteoporotic sheep model [16,18]. Several studies with other bioactive glasses have shown increases in various angiogenic markers through the direct and indirect contact of the relevant cells with the particles of these materials or their dissolution products, confirming their ability to stimulate neovascularization [64,65,66]. We hypothesized that the calcium ions released from MBG-75S could be playing a pivotal physiological modulator role in the angiogenic response of these cells through a calcium-sensing receptor, as previously reported for bone marrow-derived Flk-1^+^ CD34^+^ progenitor cells [67]. Moreover, this increase in VEGFR2 expression could also be related to local acidity effects linked to ion release from MBG-75S, as such pH conditions that have been reported to prompt VEGF-induced vasculogenesis in bone-marrow EPCs [68].

### 3.7. Action of Ion Release from MBG-75S on VEGFR2 and CD206 Expression in Cocultured EPCs and RAW 264.7 Macrophages, Respectively

Since experimental coculture models are closer to physiological conditions [8,9], and in order to know the possible modulatory role of macrophages on the action of ion release from MBG-75S on VEGFR2 expression, we carried out the coculture of EPCs with RAW 264.7 macrophages, as described in Section 2.12 and compared the action of ion release from MBG-75S on EPCs in monoculture and in coculture with these macrophages. Additionally, 1 mg/mL of MBG-75S was deposited on transwell inserts allowing the ions to diffuse through the pores in both the monocultures (EPC MBG T) and cocultures (EPC RAW MBG T). In addition, cocultures of EPCs with RAW 264.7 macrophages in the absence of material (EPC RAW) and cocultures of EPCs with angiogenic M2d macrophages in the absence of material (EPC RAW M2d) were carried out in parallel.

**Figure 9:**
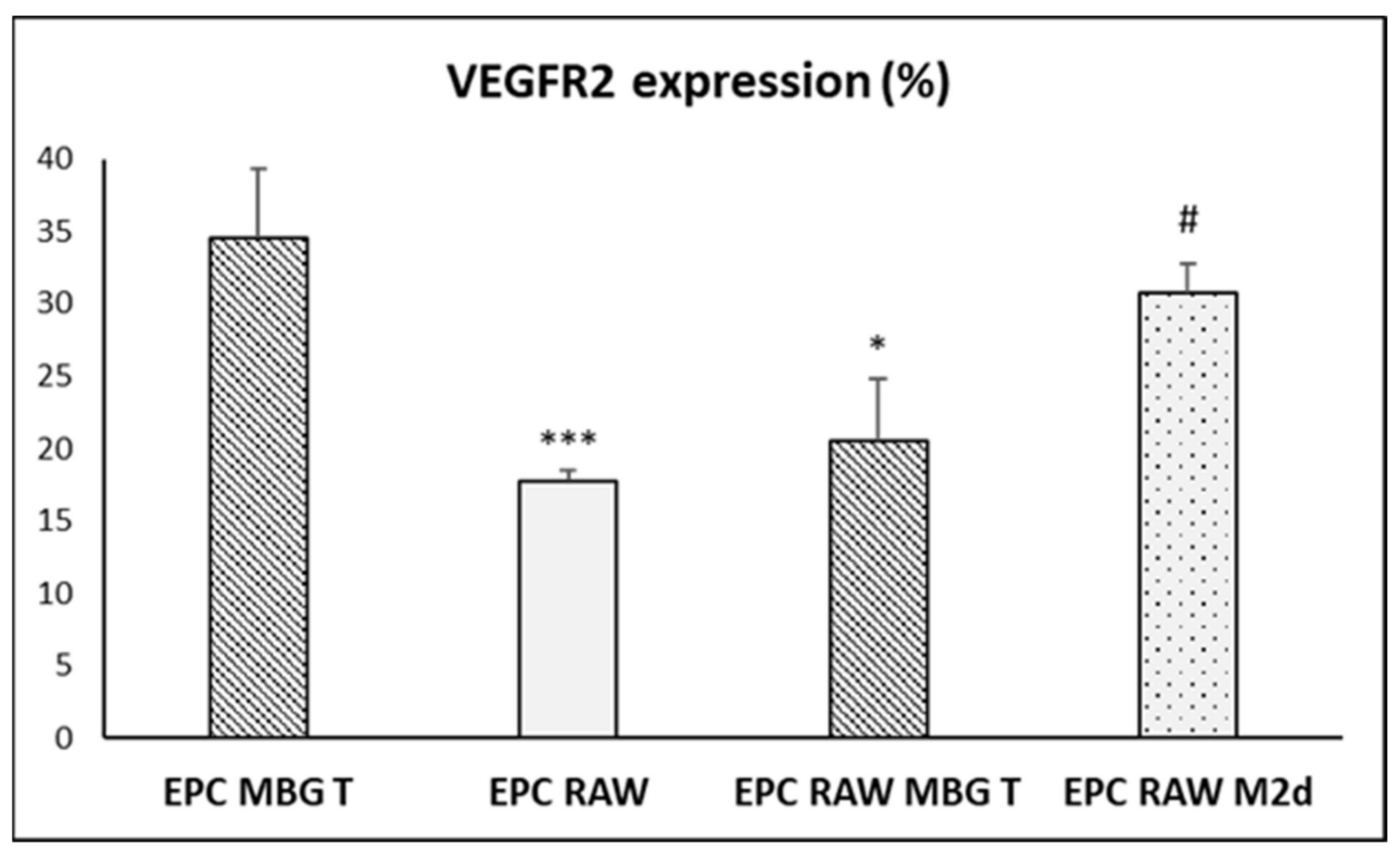
Comparison of the action of ion release from MBG-75S on VEGFR2 expression in endothelial progenitor cells (EPCs) in monoculture (EPC MBG T) and in coculture with RAW 264.7 macrophages (EPC RAW MBG T). For the coculture, RAW 264.7 macrophages were seeded on glass coverslips introduced into 6-well culture plates and EPCs were seeded around each coverslip. Additionally, 1 mg/mL of MBG-75S was deposited on transwell inserts, allowing the ions to diffuse through the pores in both monocultures and cocultures. VEGFR2 expression was evaluated as a specific marker of in vitro angiogenesis after 7 days of culture. Cocultures of EPCs with RAW 264.7 macrophages in the absence of material (EPC RAW) and cocultures of EPCs with RAW M2d macrophages in the absence of material (EPC RAW M2d) were carried out in parallel. Statistical significance: * *p* < 0.05, *** *p* < 0.005 (comparison with EPC MBG T); # *p* < 0.05 (comparison with EPC RAW).

As it can be observed in Figure 9, EPCs cocultured with RAW 264.7 macrophages in the absence of material (EPC RAW) showed much lower values of VEGFR2 expression than those obtained in EPC monoculture and treated with 1 mg/mL of MBG-75S deposited on transwell inserts (EPC MBG T). However, in the presence of both factors (EPC RAW MBG T), the VEGFR2 expression obtained is in an intermediate range. Taking into account the inductive effect observed in Figure 8, the results in Figure 9 suggest an inhibitory action of macrophages on angiogenesis in vitro, compared to the positive inductive effect produced by the ions released from the material. Cocultures of the EPCs with angiogenic RAW M2d macrophages (obtained as described in Section 2.12) in the absence of material (EPC RAW M2d) were also carried out. As it can be observed in Figure 9, the VEGFR2 expression was higher in the presence of these M2d macrophages (EPC RAW M2d) than in the presence of the control macrophages (EPC RAW); this demonstrates the sensitivity of this experimental coculture model and shows the different effect of macrophages, depending on their phenotype and activation state [23,24].

**Figure 10:**
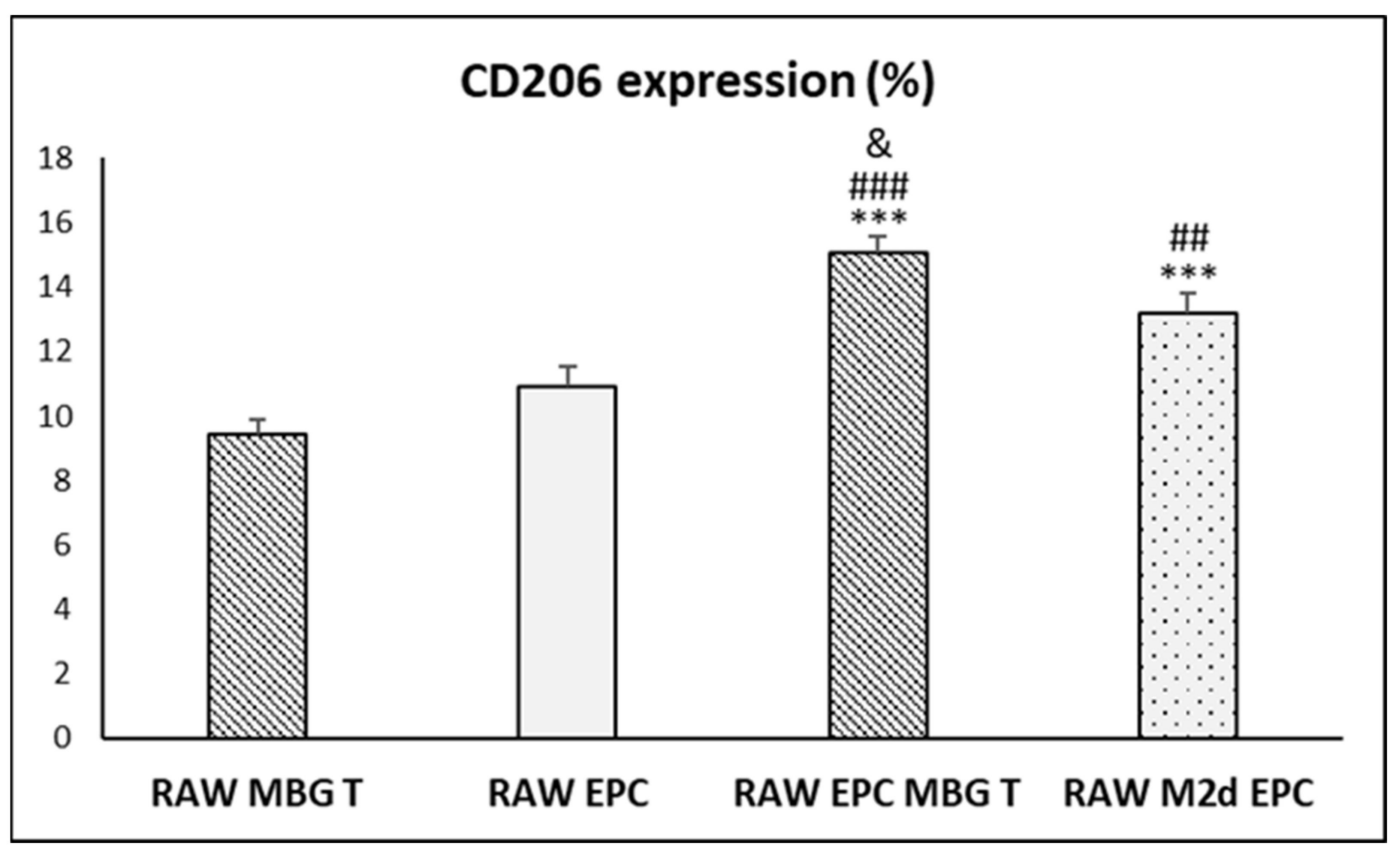
Comparison of the action of ion release from MBG-75S on CD206 expression in RAW 264.7 macrophages in monoculture (RAW MBG T) and in coculture with EPCs (RAW EPC MBG T). For the coculture, RAW 264.7 macrophages were seeded on glass coverslips introduced into 6-well culture plates and EPCs were seeded around each coverslip. Additionally, 1 mg/mL of MBG-75S was deposited on transwell inserts, allowing the ions to diffuse through the pores in both monocultures and cocultures. CD206 expression was evaluated as specific marker of M2 phenotype after 7 days of culture. Cocultures of RAW 264.7 macrophages with EPCs in the absence of material (RAW EPC) and cocultures of RAW M2d macrophages with EPCs in the absence of material (RAW M2d EPC) were carried out in parallel. Statistical significance: *** *p* < 0.005 (comparison with RAW MBG T); ### *p* < 0.005, ## *p* < 0.01 (comparison with RAW EPC); & *p* < 0.05 (comparison with RAW M2d EPC).

On the other hand, we also carried out the comparison of the action of ion release from MBG-75S on CD206 expression in RAW 264.7 macrophages in monoculture (RAW MBG T) and in coculture with EPCs (RAW EPC MBG T). Figure 10 shows that the ions released by MBG-75S induced a significant increase on CD206 expression in macrophages cocultured with EPCs (RAW EPC MBG T), in comparison with macrophages in monoculture in the presence of MBG deposited on transwells (RAW MBG T), in comparison with macrophages cocultured with EPCs in the absence of material (RAW EPC), and in comparison with angiogenic M2d macrophages cocultured with EPCs in the absence of material (RAW M2d EPC). On the other hand, the values of CD206 expression in these M2d macrophages cocultured with EPCs in the absence of material (RAW M2d EPC) were also higher than the values obtained in macrophages in monoculture in the presence of MBG deposited on transwells (RAW MBG T) and in macrophages cocultured with EPCs in the absence of material (RAW EPC). These results indicate that the ions released by MBG-75S induced a significant increase of this specific marker of M2 macrophage phenotype [24] in macrophages cocultured with EPCs, and this value was even higher than that obtained in angiogenic M2d macrophages cocultured with EPCs. This significant impact of the presence of EPCs on the CD206 expression in macrophages is in agreement with their known role in the creation of an instructive niche for the polarization of M2-like macrophages [69]. The increase in CD206^+^ cells has recently been related to an accelerated remodeling response by M2 reparative macrophages, which accompanies bone repair [70]. This result suggests the possible role of M2 macrophages in bone repair induced by MBG-75S.

## 4. Conclusions

The present study demonstrates that ions released by MBG-75S, with the molar composition 75SiO_2_-20CaO-5P_2_O_5_, promote the differentiation of MC3T3-E1 pre-osteoblasts and induce the VEGFR2 expression in EPCs, highlighting their potential action on osteogenesis and angiogenesis. Moreover, these ions were able to attenuate the inhibitory effect produced on both processes by macrophages cocultured with these cell types. In addition, the ions released by MBG-75S also induced a significant increase on CD206 expression in macrophages cocultured with EPCs, suggesting the role of reparative M2 macrophages in bone repair induced by MBG-75S and demonstrating the high potential of this biomaterial for the preparation of implants for bone regeneration.

## Figures and Tables

**Table 1:** MBG-75S estimated oxides composition measured by X-ray fluorescence (XRF).

Oxide	Molar %
SiO_2_	71.4
CaO	25.2
P_2_O_5_	3.4

## Data Availability

Data is contained within the article.
